# Therapeutic Effect of IL-21 Blockage by Gene Therapy in Experimental Autoimmune Encephalomyelitis

**DOI:** 10.1007/s13311-022-01279-8

**Published:** 2022-07-28

**Authors:** Ángel Edo, Laura Calvo-Barreiro, Herena Eixarch, Assumpció Bosch, Miguel Chillón, Carmen Espejo

**Affiliations:** 1 Institut de Neurociències (INc), Department of Biochemistry and Molecular Biology, Universitat Autònoma de Barcelona, (Campus UAB), Bellaterra, Cerdanyola del Vallès (Ed. H 5th level), 08193 Barcelona, Spain; 2 Vall d’Hebron Institut de Recerca, Hospital Universitari Vall d’Hebron, Barcelona, Spain; 3 Servei de Neurologia-Neuroimmunologia, Centre d’Esclerosi Múltiple de Catalunya, Vall d’Hebron Institut de Recerca, Hospital Universitari Vall d’Hebron, Vall d’Hebron Barcelona Hospital Campus, Pg. Vall d’Hebron 119-129 (Ed. Collserola, Lab. 149), 08035 Barcelona, Spain; 4 Universitat Autònoma de Barcelona, Bellaterra, Cerdanyola del Vallès, Spain; 5 Institució Catalana de Recerca I Estudis Avançats (ICREA), Barcelona, Spain; 6 Vector Production Unit (UPV), Universitat Autònoma de Barcelona, Barcelona, Spain

**Keywords:** IL-21, Soluble receptor, EAE, Multiple sclerosis, AAV

## Abstract

The pathogenic role of the interleukin 21 (IL-21) in different autoimmune diseases, such as multiple sclerosis (MS), has been extensively studied. However, its pleiotropic nature makes it a cytokine that may exhibit different activity depending on the immunological stage of the disease. In this study, we developed a gene therapy strategy to block the interaction between IL-21 and its receptor (IL-21R) by using adeno-associated vectors (AAV) encoding a new soluble cytokine receptor (sIL21R) protein. We tested this strategy in a murine model of experimental autoimmune encephalomyelitis (EAE), obtaining different clinical effects depending on the time at which the treatment was applied. Although the administration of the treatment during the development of the immune response was counterproductive, the preventive administration of the therapeutic vectors showed a protective effect by reducing the number of animals that developed the disease, as well as an improvement at the histopathological level and a modification of the immunological profile of the animals treated with the AAV8.sIL21R. The beneficial effect of the treatment was also observed when inducing the expression of the therapeutic molecule once the first neurological signs were established in a therapeutic approach with a doxycyline (Dox)-inducible expression system. All these clinical results highlight the pleiotropicity of this cytokine in the different clinical stages and its key role in the EAE immunopathogenesis.

**Supplementary Information:**

The online version contains supplementary material available at 10.1007/s13311-022-01279-8.

## Introduction

Multiple sclerosis (MS) is a chronic inflammatory and demyelinating disease of the central nervous system (CNS) mediated by the immune system. MS is the most prevalent demyelinating disease in the world, with more than 2.8 million patients worldwide [1]. Nowadays, MS immunopathogenesis is understood as a set of bidirectional interactions between the components of the adaptive and innate immune system in the periphery, which includes T cells, B cells, and myeloid cells, and resident cells of the CNS, such as microglia and astrocytes [2]. Effector CD4 T cells play an important role in MS pathogenesis and are located within inflammatory lesions of the CNS and in the cerebrospinal fluid of MS patients [3]. Initially, it was thought that the principal cause for experimental autoimmune encephalomyelitis (EAE) and MS was the induction of Th1 response by IL-12 [4]; however, the cellular subtype Th17 (the main producer of IL-17) was later identified as a key player in autoimmunity [5]. Although the encephalitogenic role has historically been attributed to autoreactive CD4 T cells, CD8 T cells are more abundant in human inflammatory lesions, and their frequency strongly correlates with axonal damage [3, 6]. On the other hand, there are components of the immune system capable of modulating the inflammation by switching to Th2 immune responses or maintaining self-tolerance (i.e., regulatory T (Treg) cells), thus generating a cycle of inflammation and its resolution [7].

There are different key cytokines involved in the pathogenesis of MS; however, several evidences have pointed to interleukin 21 (IL-21) as one of the main cytokines involved in the initiation of the immune response. IL-21 regulates the activity of both innate (monocytes/macrophages, natural killer (NK) cells, and dendritic cells (DCs)) and the adaptive immune system (T and B cells). Although IL-21 can exert certain suppressive functions on immunity, it is mainly known for its broad capacity to stimulate the proliferation and activity of pro-inflammatory pathways involved in the immunopathogenesis of MS and other autoimmune diseases such as rheumatoid arthritis (RA) or systemic lupus erythematosus (SLE) [8, 9]. The functions of IL-21 include the differentiation and proliferation of pathogenic Th17 cells [10, 11], the increase of follicular T helper cell proliferation [12], the stimulation of cytotoxicity and proliferation of CD8 T, NK, and NKT cells [8, 13], as well as the inhibition of the differentiation and survival of Treg cells [14]. Various genetic studies have established a relationship between the polymorphisms detected at the IL-21 receptor (IL-21R) locus and the susceptibility to develop MS [15]. In addition, histopathological studies of CNS lesions of patients in the acute or chronic phases of MS have shown that both IL-21 and IL-21R are expressed on CD4 T cells, and the latter has also been detected on the cortical neurons of MS lesions [16]. These observations correlate with a higher expression of IL-21 mRNA during inflammatory relapses [17] and the detection of a high number of IL-21-producing CD4 T cells in the CSF of patients in the progressive stage [18]. Also, patients treated with Alemtuzumab (anti-CD52 treatment) have decreased levels of IL-21 in serum [19]. All these highlights expose the involvement of IL-21 in the inflammatory response that occurs in MS.

In the present work, we have studied the effect of IL-21 sequestration in different clinical stages by administering adeno-associated vectors (AAV) encoding a soluble IL-21R (sIL21R) capable of selectively blocking the function of IL-21 in an EAE mouse model of MS. Similar strategies have been tested in other inflammatory diseases such as SLE or RA, obtaining promising results [9]. Our results show that the preventive treatment with sIL21R produced a clinical improvement due to a reduction in the incidence of the disease, as well as an improvement in the histopathological signs of the CNS. On the contrary, the blockade of IL-21 during the development of the immune response (before the onset of clinical signs) was counterproductive, increasing EAE clinical severity. Additionally, treatment with inducible AAV vectors that allowed sIL21R expression once the first neurological symptoms were stablished, led to a clinical improvement. All these results reveal the pleiotropic role of IL-21 in the different clinical and immunological stages of EAE and its interest as a therapeutic target for the treatment of MS.

## Methods

### Viral Vector Production and Purification

AAV8 and adenovirus 5 (Ad5) vectors were produced, purified, and manipulated in the biosafety level 2 facilities at the Universitat Autònoma Barcelona. Briefly, AAV8 vectors were generated using the triple transfection system in HEK293 cells. After 48 h, AAV vectors were harvested, treated with benzonase (71,206–3, Merck, Kenilworth, NJ, USA), purified in an iodixanol gradient, and titrated using the Picogreen system [20]. To produce Ad5 vectors, PacI-linearized plasmids were transfected into HEK293 cells, and the virus was recovered 8 to 10 days post-transfection. Then, viruses were sequentially amplified, purified by CsCl gradients, and infective particles measured by an endpoint dilution assay that counted the number of hexon-producing cells in triplicate using the AdEasy Viral Titer Kit (972,500, Agilent Technologies, Santa Clara, CA, USA) [21]. All Ad5 vectors used in the in vitro experiments contained CMV promoter. While AAV8 vectors used in the in vivo experiments with the animal model contained CMV, CAG or TetOn-CMV promoter depending on the therapeutic approach.

### Immunocytochemistry and Immunodetection of sIL21R

HEK293 cells were infected with a multiplicity of infection (MOI) of 2 or 5 for 24–48 h with Ad5.CMV.sIL21R and Ad5.CMV.Null or Ad5.CMV.eGFP as negative controls. For immunocytochemistry, anti-IL21R antibody (AF596, R&D Systems, Minneapolis, MN, USA) and Alexa Fluor 488 donkey anti-goat secondary antibody (A11055, Invitrogen, Carlsbad, CA, USA) were used at a dilution of 1:100 and 1:200, respectively. For immunodetection, cell pellet was homogenized in lysis buffer (RIPA) and total protein was determined from lysed cells and supernatants using the Pierce BCA Protein Assay (232,227, Thermo Fisher Scientific, Waltham, MA, USA). Protein extracts and supernatants were loaded onto denaturing acrylamide gels and then electrotransferred to PDVF membranes (10,600,023, GE Healthcare, Chicago, IL, USA). Anti-IL21R primary antibody (AF596, R&D Systems) was used at 1:1000 dilution and goat HRP-anti-IgG secondary antibody (P0160, Dako, Agilent Technologies, Santa Clara, CA, USA) at 1:10,000 were used in the presence of 5% (w/v) of BSA (A9418, Sigma-Aldrich, St. Louis, MO, USA).

### In Vitro Blocking of Murine IL-21

sIL21R and eGFP conditioned media were produced in HEK293 cells by the infection with Ad5.sIL21R, and Ad5.eGFP as a control, using a MOI of 10. Cells were harvested 30 h post-infection, lysed by freeze–thaw cycles, centrifuged 10 min at 3000 rpm to obtain the cleared supernatants, and stored at −80 °C until use. Fresh splenocytes were cultured in 6-well plates at 6·10^6^ cells/well, in DMEM supplemented with 10% FBS (1,237,980, Thermo Fisher Scientific). Recombinant murine IL-21 (12,340,212, Immunotools GmbH, Frieshoythe, Germany) at 10 ng/mL was incubated for 1 h at 37 °C in sIL21R or eGFP conditioned culture media, and latter, added to the splenocytes for 15 min. Then, splenocytes were harvested, medium discarded by centrifugation, and cells resuspended in lysis buffer (RIPA). Results were analyzed by Western blot using anti-pY705-Stat3 (9145S), anti-Stat3 (4904S), and anti-Gapdh (2118S; Cell Signaling Technology, Danvers, MA, USA) as primary antibodies, all at 1:1000 dilution. Rabbit HRP-anti-IgG secondary antibody (P0399; Dako) at 1:10,000 dilution was used in the presence of 5% (w/v) BSA. Band intensities were quantified using Image Lab (Bio-Rad Laboratories, Hercules, CA, USA).

### Animals and Ethical Permissions

Female (6- to 9-week-old) C57BL/6JOlaHsd mice were purchased from Envigo (Venray, The Netherlands) and housed under standard laboratory conditions. All procedures were performed in accordance with European Union (Directive 2010/63/EU) and governmental regulations (Real Decreto 53/2013; Generalitat de Catalunya Decret 214/97). The Ethics Committee on Animal Experimentation of the Vall d´Hebron Research Institute approved all procedures described in this study (protocol number: 9533 CEEA).

### Induction of EAE and Clinical Follow-Up

Anesthetized mice were immunized by subcutaneous injections of 100 μg MOG_40-55_ (Proteomics Section, Universitat Pompeu Fabra, Barcelona, Spain) emulsified in 100 μL of CFA (IFA (F5506, Sigma-Aldrich) containing 4 mg/mL *Mycobacterium tuberculosis* H37RA (231,141, BD Biosciences, San Jose, CA, USA)). At 0 and 2 days postimmunization (p.i), mice were intravenously (i.v.) injected with 250 ng of pertussis toxin (P7208, Sigma-Aldrich). The clinical symptoms of EAE were monitored daily using the following criteria: 0, no clinical signs; 0.5, loss of caudal tail tone; 1, paralysis of whole tail; 2, mild paraparesis of one or both hindlimbs; 2.5, severe paraparesis or paraplegia; 3, mild tetraparesis; 3.5, moderate tetraparesis; 4, tetraparesis (paralyzed hindlimbs); 4.5, quadriplegia (paralyzed forelimbs); 5, moribund; and 6, death. Corrective measures and endpoint criteria to ensure EAE-incident animals’ welfare were applied as described in [22]. All data presented are in accordance with the ARRIVE guidelines for animal research and with the guidelines suggested for EAE publications [23].

### In Vivo AAV and Doxycycline Administration

To determine the prophylactic effect of sIL21R expression in EAE mice, a single dose of 5·10^11^ vg/mouse of AAV8 vectors (Null or sIL21R) was administered 21 days before EAE induction. This dose was determined on the basis of a previous study done by our group [25]. In the early therapeutic experiment, a dose of 10^12^ viral genomes (vg)/mouse of AAV8 vectors was administered at days −1 or +1 p.i. The null control is a vector that has the same serotype as the therapeutic vector but does not encode any transgene as it contains a non-transcribed DNA. Finally, in the therapeutic approach with doxycycline (Dox)-inducible vectors, a dose of 10^12^ vg/mouse was administered 15 days before EAE induction and the expression of the inducible system was daily activated with an intraperitoneal (i.p.) administration of 25 mg/kg Dox-Hyclate (D9891, Sigma-Aldrich) diluted in 100 μL of NaCl 0.9% from the day on which the mice had a clinical score of 0.5. In all experiments, the viral dose was diluted in 200 μL of NaCl 0.9% and was administered by i.v. injection through the lateral tail vein.

### Splenocytes Proliferative Assay

Splenocyte suspensions were obtained by griding murine spleens through a 70 μm nylon cell strainer at 27 days p.i. In all proliferative assays, 2·10^5^ splenocytes/well were seeded in 96-well plates in 200 μL of proliferation medium (X-VIVO™ 15 medium (BE02-060F, Lonza, Basel, Switzerland) supplemented with 1% (v/v) L-glutamine (X0550, Biowest, Nuaillé, France), 0.4% (v/v) penicillin–streptomycin (L0022, Biowest), 0.1 M HEPES (H0887, Sigma-Aldrich), and 6 μM 2-β-mercaptoethanol (M3148, Sigma-Aldrich)). Cells were stimulated with 5 μg/mL of MOG_40-55_ or 5 μg/mL of phytohemagglutinin (PHA-L) (L2769, Sigma-Aldrich) and cells without any stimulus were used as baseline controls. After 48 h, 75 μL of supernatant were harvested and stored at −80 °C to further assess cytokine secretion. At the same time, 1 μCi/well of [^3^H]-thymidine (NET027Z, PerkinElmer, Waltham, MA, USA) was added and incubated for 18 h. The levels of radioactivity were measured using a β-scintillation counter (Wallac, Turku, Finland). Five replicates per mouse and condition (control, MOG_40-55_, PHA-L) were performed and results were expressed as the stimulation index. Stimulation index was calculated by dividing the mean counts per minute of MOG_40-55_ or PHA-L condition by the mean of the control condition.

### Cytokine Detection

Cytokine secretion was determined in the supernatants of MOG_40-55_ stimulated splenocytes using the Th1/Th2/Th9/Th17/Th22/Treg Cytokine 17-Plex Mouse ProcartaPlex Panel (EPX170-26,087–901, Invitrogen) and TGF beta 1 Mouse Procartaplex Simplex Kit (EPX01A-20608–901, Invitrogen), according to the manufacturer’s instructions. Data were analyzed with a Magpix instrument (Luminex Corporation, Austin, TX, USA) and ProcartaPlex Analyst software (Thermo Fisher Scientific).

### Analysis of mRNA Levels by qRT-PCR

Total RNA was extracted from the isolated splenocytes using the RNeasy Mini Kit (74,104, Qiagen), treated with DNase I Amplification Grade (AMPD1, Sigma-Aldrich) and reverse transcribed with iScript cDNA Synthesis Kit (1,708,891, Bio-Rad Laboratories). Primers for *Tgfb1* (Mm01178820, Applied Biosystems, Foster City, CA, USA), *Rorc2* (custom assay as described in [24]), *Tbx21* (Mm00450960, Applied Biosystems), *Foxp3* (Mm00475162, Applied Biosystems), *Gata3* (Mm00484683, Applied Biosystems), *Il4* (Mm00445259, Applied Biosystems), *Il10* (Mm01288386, Applied Biosystems), *Ifng* (Mm01168134, Applied Biosystems), *Il17a* (Mm00439618, Applied Biosystems), and *Gapdh* (Mm99999915, Applied Biosystems) as well as TaqMan Gene Expression Master Mix (4,369,016, Applied Biosystems) were used to perform qPCR according to the manufacturer’s instructions. All experiments were performed on CFX384 Touch Real-Time PCR Detection System (Bio-Rad Laboratories) and the analysis of qPCR output data followed the manufacturer-suggested 2^−ΔΔCt^ method [26]. The expression of the housekeeping gene (*Gapdh*) was used to normalize the expression of the different genes of interest while the Null group was used as a calibrator.

### Histological Studies

At the endpoint of the experiment (day 27 p.i), mice were euthanized, and spinal cords collected and fixed in 4% paraformaldehyde at 4 °C for 24 h, embedded in paraffin, and cut into 4-μm-thick transversal sections. Samples were immunostained to evaluate several histopathological parameters: demyelination with anti-myelin basic protein (MBP) antibody (AB980, Merck), axonal damage with anti-neurofilament H, non-phosphorylated antibody (SMI-32) (801,701, Biolegend, San Diego, CA, USA), CD3^+^ infiltrating cells with anti-CD3 antibody (A0452, Dako) and reactive astroglia with anti-glial fibrillary acidic protein (GFAP) antibody (Z0334, Dako). For reactive microglia identification, lectin from *Lycopersicon esculentum* (LEA) (L0651, Sigma-Aldrich) was used. Briefly, samples were deparaffinized and rehydrated, and antigen retrieval (SMI-32 and CD3 stainings) and incubation with blocking solution for 1 h at room temperature were performed. Then, primary antibodies diluted in blocking solution were incubated overnight at 4 °C. After several washes, the samples were incubated 1 h at room temperature with their respective secondary antibodies diluted in blocking solution. Finally, cell nuclei were stained with 4’, DAPI (D9542, Sigma-Aldrich). Images were taken using a Nikon Eclipse 90i fluorescence microscope (Nikon, Minato, Japan) and quantification performed with the ImageJ analysis software (Wayne Rasband National Institute of Health, Bethesda, MD, USA). Regarding anti-CD3 labelling, positive cells were counted manually and the results expressed as CD3^+^ cells/mm^2^ of the entire white matter area. Quantifications of MBP, LEA, SMI-32, and GFAP labelling are expressed as the percentage of the positive area respect to the entire white matter area. In all cases, two transversal sections of caudal spinal cord per animal were analyzed.

### Flow Cytometry

To determine the immunological profile, spleen cell suspensions were prepared as described previously. First, dead cells were labelled with Fixable Viability Stain (BD Pharmigen, BD Biosciences) and cells were blocked with anti-mouse CD16/32 (553,142, BD Pharmigen). To analyse Treg cell population, antibodies against CD3 $\varepsilon$ (561,827, BD Pharmigen), CD4 (12–0251, eBiosciences), CD8a (563,152, BD Horizon), CD25 (12–0251, eBiosciences), and Foxp3 (17–5773, eBiosciences) were used. For intracellular labelling of Foxp3, the Foxp3 Fixation/Permeabilization Kit (00–5523-00, eBiosciences) was used following the manufacturer’s instructions. For B cell subsets, antibodies against B220 (552,772, BD Pharmigen), CD1d (553,846, BD Pharmigen), CD5 (550,035, BD Pharmigen), CD19 (560,143, BD Pharmigen), CD138 (562,610, BD Horizon), and MHCII (562,363, BD Pharmigen) were used. For DC subpopulations and activation status, antibodies against B220 (553,088, BD Pharmigen), CD11b (25–0112, eBiosciences), CD11c (47–0114, eBiosciences), CD8a (563,152, BD Horizon), CD80 (553,769, BD Pharmigen), and MHCII (562,363, BD Pharmigen) were used. For analysis of macrophage, antibodies against CD11b (562,605, BD Horizon), CD206 (141,712, BioLegend), F4/80 (565,410, BD Pharmigen), Ly6C (560,593, BD Pharmigen), and Ly6G (551,460, BD Pharmigen) were used. For NK cells and its activation status, antibodies against CD3*ε* (561,827, BD Pharmigen), CD69 (560,689, BD Pharmigen) and NK1.1 (563,220, BD Horizon) were used. For the analysis of cytokine production, splenocytes were stimulated ex vivo with 50 ng/mL PMA (P1585, Sigma-Aldrich) and 1 μg/mL ionomycin (I0634, Sigma-Aldrich) in the presence of GolgiPlug (555,029) and GolgiStop (554,724, BD Bioscences) for 6 h. Extracellular staining with anti-CD3*ε* (561,827, BD Pharmigen), anti-CD4 (46–0042, eBiosciences), and anti-CD8a (563,152, BD Horizon) was performed. Intracellular staining of cytokines was performed to detect IL-17A (561,020, BD Pharmigen), IFNγ (563,376, BD Horizon), IL-4 (554,436, BD Pharmigen), and IL-10 (563,276, BD Horizon). Anti-CD69 (560,689, BD Pharmigen) was used as control of proper ex vivo stimulation. For intracellular staining, BD Cytofix/Cytoperm Fixation/Permeabilization Kit (554,714, BD Biosciences) was used according to manufacturer’s instructions. Fluorescence minus one controls were included in the analysis to properly interpret the data. Samples were acquired in a CytoFLEX flow cytometer and data was analyzed with the CytExpert 2.3 software (Beckman Coulter, Brea, CA, USA).

### Statistical Analysis

The data on the text are expressed as the mean of the values obtained ± the standard deviation of the mean (SD), while the graphs are represented as the mean of the values ± the standard error of the mean (SEM). When the data of the groups to be compared followed a normal distribution, parametric analyses were applied. When comparing two independent groups, Student *t* analysis was used, while for the comparison of three or more groups, ANOVA test with a Tukey post hoc test was used. Nonparametric analyses were applied when the data did not have a normal distribution. Mann–Whitney analysis was used for the comparison of two independent groups, while the Kruskal–Wallis test with a Dunn’s post hoc test was used for the comparison of three or more groups. Statistical analysis of the data was performed with SPSS 26.0 (IBM, New York, NY, USA) and GraphPad Prism 8.0 (GraphPad Holdings, San Diego, CA, USA), which was also used to generate the graphs. Bilateral hypothesis analysis was applied in all cases and differences were considered statistically significant when the *p* value was < 0.05.

## Results

### Design and Characterization of sIL21R

**Fig. 1:**
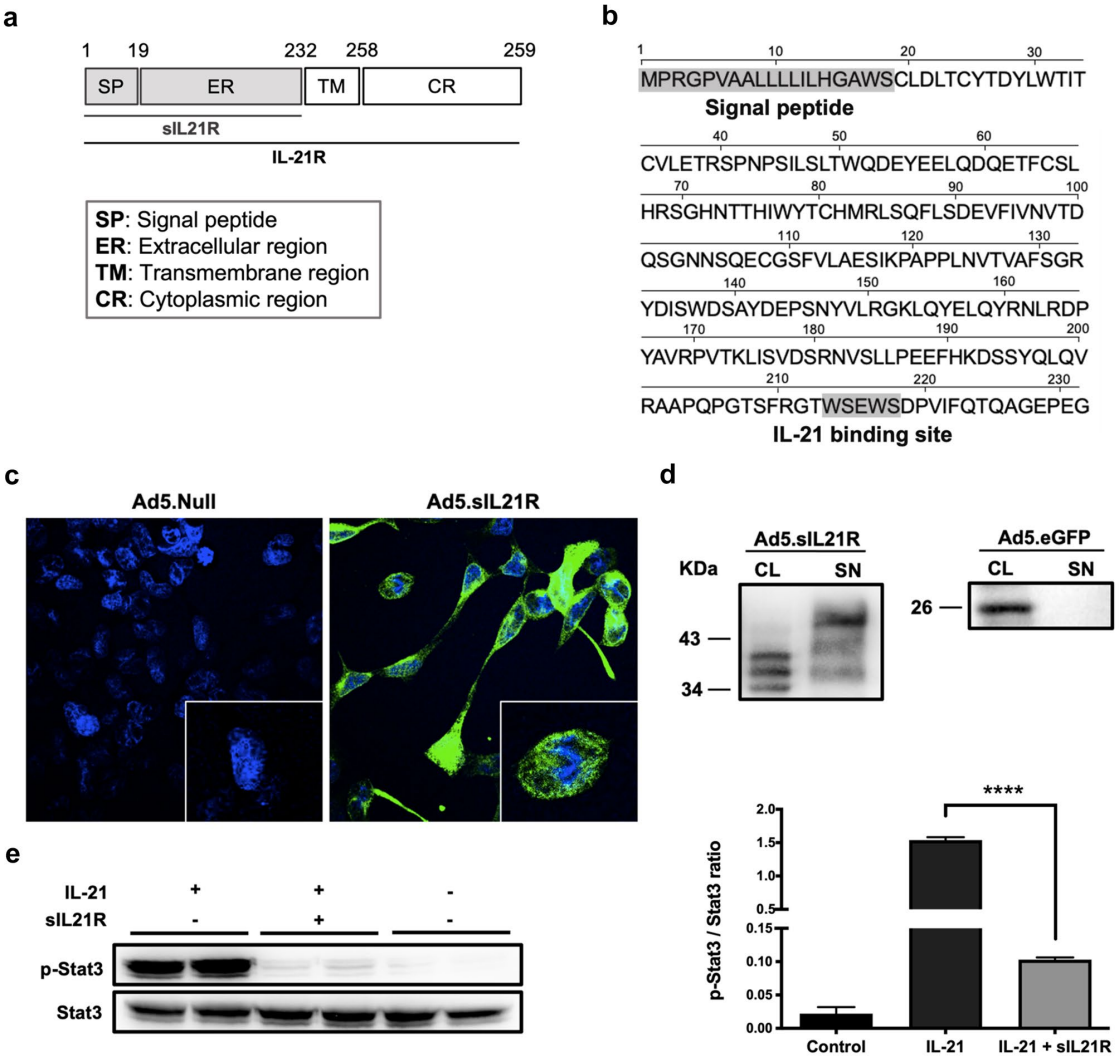
Biochemical and functional characterization of the sIL21R. **a** Schematic representation of the transmembrane IL-21R and the designed sIL21R (in gray). **b** Sequence of the therapeutic molecule, that contains the secretion signal and the extracellular region containing the described IL-21 binding site. **c** Immunocytochemistry with anti-IL-21R antibody (green) reveals cellular distribution of the soluble receptor in HEK293 cells infected with Ad5.sIL21R. Ad5.Null-infected cells were used as a negative control. Cell nuclei were stained with Hoechst (blue). **d** Secretion of sIL21R into the extracellular medium was detected in the supernatants (SN) of HEK293 cells infected with Ad5.sIL21R. sIL21R was also detected in the cell lysate (CL). Ad5.eGFP-infected HEK293 cells were used as a negative secretion control. Representative Western blot of three independent experiments with primary anti-IL-21R and anti-eGFP antibody. **e** sIL21R blocked IL-21-Stat3 phoshorylation signalling by inhibiting its binding to IL-21R. Mouse primary splenocytes were incubated for 15 min with 10 ng/mL of IL-21 that had previously been co-incubated with or without sIL21R. Control: unstimulated splenocyte cell lysate. Representative Western blot and quantification of the pStat3/Stat3 ratio from two independent experiments. Data are represented as mean ± SEM. ****p < 0.0001

sIL21R was designed based on the transmembrane receptor (NCBI GenBank NM_021887.2) (Fig. 1a), since no natural form of the soluble receptor has been identified, neither human nor murine. However, only the extracellular region of the IL-21R was used as a therapeutic molecule, which contains both the secretion signal and the canonical binding site for IL-21 (Fig. 1b).

First, a complete characterization of the sIL21R was carried out to demonstrate that the recombinant protein was soluble, secretable, and capable of selectively blocking murine IL-21. HEK293 cells were infected with Ad5.sIL21R or Ad5.Null (control) with a low MOI (MOI = 2) to avoid saturation. Immunocytochemistry of the infected cells revealed a clear dispersed cytoplasmic localization of sIL21R (Fig. 1c), which confirmed the solubility of sIL21R. To assess its secretion capacity, HEK293 cells were infected with Ad5.sIL21R or Ad5.eGFP, as a negative control of secretion, using a higher MOI (MOI = 5) to favor its detection. After 24 h, sIL21R was detected in the cell culture medium, suggesting that the therapeutic molecule is highly secretable (Fig. 1d).

When natural IL-21 binds to its membrane receptor, it signals through the phosphorylation of Stat3. In a preliminary experiment with mouse primary splenocytes, we determined that an incubation time of 15 min with IL-21 was sufficient to detect the phosphorylated form of Stat3 (p-Stat3). In order to demonstrate that sIL21R blocks IL-21 signalling pathway, IL-21 and sIL21R were co-incubated for 1 h and then added to splenocytes. As a result, we observed a 90–95% reduction of the p-Stat3 signal (Fig. 1e).

### AAV8.CMV.sIL21R Treatment Prevents the Development of EAE

**Fig. 2:**
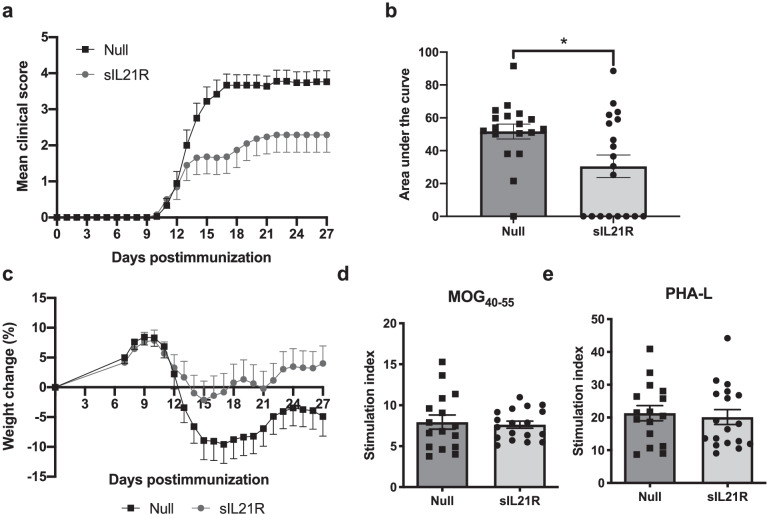
The preventive treatment with AAV8.CMV.sIL21R protects against the development of EAE. Mice were immunized with MOG_40-55_ and treated with 5·10^11^ vg/mouse of AAV8.CMV.sIL21R or AAV8.CMV.Null, 21 days prior to the induction of EAE. **a** Mean daily clinical score for each group. **b** sIL21R-treated group showed a statistically significant milder clinical course of the disease compared with the Null group. **c** Average daily weight changes relative to initial weight on the day of EAE induction. The graphs represent the combined results of two independent experiments (Null, *n* = 18; sIL21R, *n* = 19). Treatment did not alter the (**d**) antigen-specific response (MOG_40-55_) or the (**e**) polyclonal response (PHA-L) in splenocytes. The graphs represent the combined results of two independent experiments (Null: *n* = 16; sIL21R: *n* = 18). Data are represented as mean ± SEM. *p < 0.05

Once the biochemical and functional characteristics of sIL21R were assessed, we studied its therapeutic potential in an experimental model of MS, starting with a preventive approach. AAV8.CMV.sIL21R and AAV8.CMV.Null vectors were administered intravenously 21 days before the induction of EAE with a viral dose of 5·10^11^ vg per animal. As seen in Fig. 2a, sIL21R-treated group presented a significantly lower area under curve (AUC) of the clinical score (Null: 51.67 ± 19.27, *n* = 18; sIL21R: 30.49 ± 30.08, *n* = 19; *p* = 0.015) (Fig. 2b), and a lower maximum clinical score (Null: 3.97 ± 1.33, *n* = 18; sIL21R: 2.37 ± 2.17, *n* = 19; *p* = 0.02) due to a significant reduction in the number of animals which developed EAE [Null: 17/18 (94.44%); sIL21R: 11/19 (57.89%); *p* = 0.019]. The motor dysfunction of EAE is associated with weight loss, which is more pronounced in the acute phase of the disease, reaching a partial recovery that is maintained in the chronic phase. As observed in Fig. 2c, sIL21R-treated mice showed a tendency to lose less weight than the control group, however, the differences did not reach statistical significance (AUC of weight change for Null: −56.37 ± 197.00, *n* = 18; sIL21R: 48.23 ± 199.60, *n* = 19; *p* = 0.118). At the end of the experiment, strong sIL21R expression in liver was confirmed in all animals (data not shown). However, when analyzing the clinical variables of the incident mice only, the therapeutic group did not show less AUC of the clinical score than the control group (Null: 54.71 ± 14.76, *n* = 17; sIL21R: 52.66 ± 18.56, *n* = 11; *p* = 0.826).

To find out whether the treatment had any effect on T-cell reactivity to the MOG, proliferative assays of splenocytes were performed with a specific response against MOG_40-55_ and polyclonal immune response with PHA-L, as positive control. No differences were observed in the proliferative capacity of splenocytes from sIL21R and Null group neither when stimulated with MOG_40-55_ (Null: 7.94 ± 3.46, *n* = 16; sIL21R: 7.63 ± 1.84, *n* = 18; *p* = 0.740), or with PHA-L (Null: 21.31 ± 9.23, *n* = 16; sIL21R: 20.12 ± 9.61, *n* = 18; *p* = 0.716) (Fig. 2d, e). However, all splenocytes from sIL21R-treated mice presented a MOG-specific proliferation, demonstrating that mice that did not develop EAE clinical symptoms developed a proper antigen-specific T cell response due to the immunization.

### sIL21R-Preventive Treated Mice Present an Improvement in the CNS Pathology

**Fig. 3:**
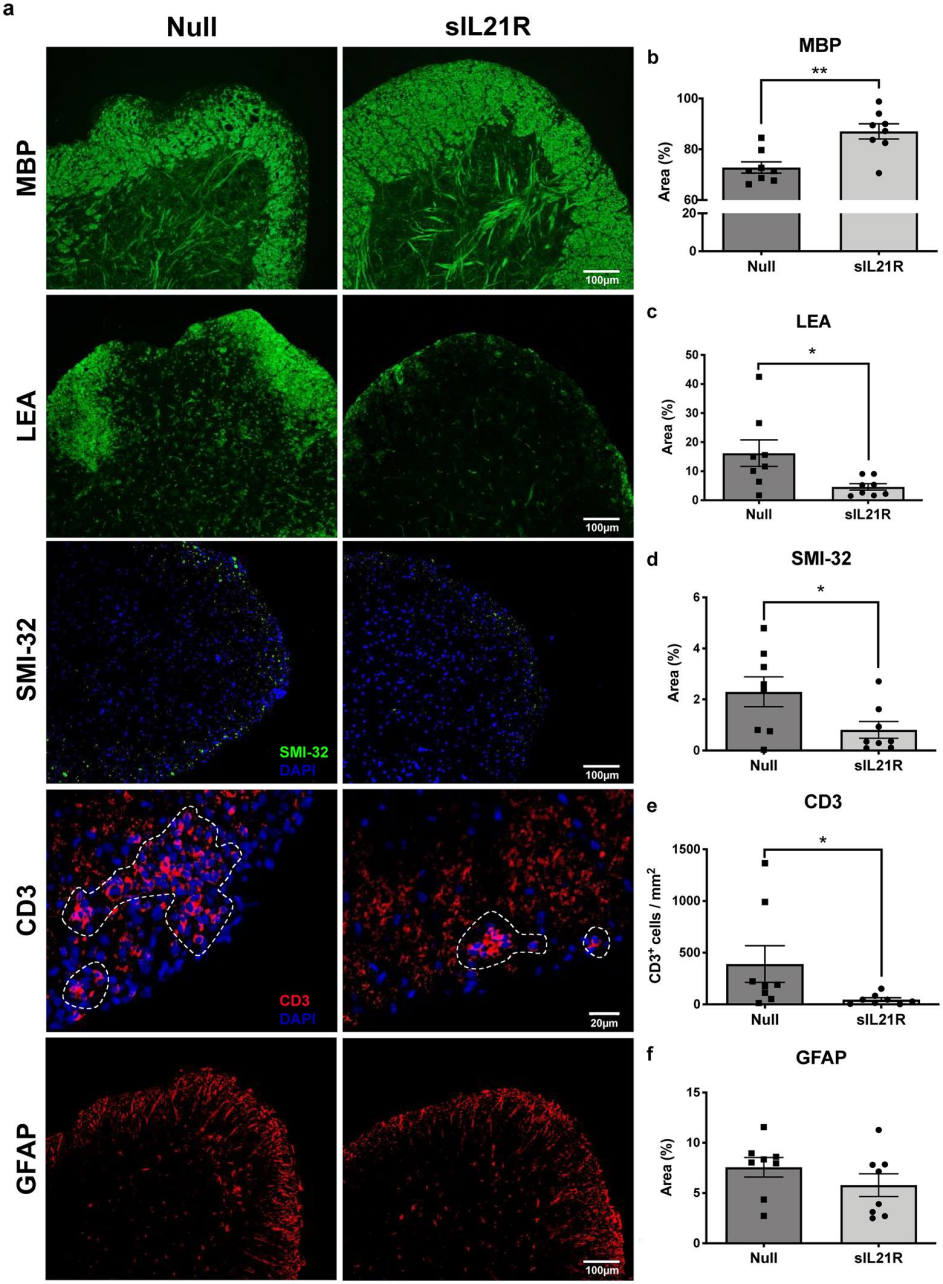
Mice treated with AAV8.CMV.sIL21R have a better CNS histopathological status. **a** Selected images from a representative animal of each group. **b** Demyelination, **c** microglia activation, **d** axonal damage, and **e** T-cell infiltration was significantly reduced in mice treated with AAV8.sIL21R, while there were no differences in **f** the GFAP marking. The CD3.^+^ T-cell infiltrate has been delimited with a dotted line. (Null: *n* = 8; sIL21R: *n* = 8). Data are represented as mean ± SEM. *p < 0.05, **p < 0.01

The mice that were preventively treated with AAV8.sIL21R presented less demyelination (Null: 72.83% ± 6.27%, *n* = 8; sIL21R: 87.05% ± 8.49%, *n* = 8; *p* = 0.002) (Fig. 3b), microglia activation (Null: 16.19% ± 12.90%, *n* = 8; sIL21R: 4.58% ± 3.14%, *n* = 8; *p* = 0.026) (Fig. 3c), axonal damage (Null: 2.30% ± 1.66%, *n* = 8; sIL21R: 0.81% ± 0.92%, *n* = 8; *p* = 0.043) (Fig. 3d) and T-cell infiltration (Null: 390.20 cells/mm^2^ ± 501.4 cells/mm^2^, *n* = 8; sIL21R: 47.21 cells/mm^2^ ± 49.65 cells/mm^2^, *n* = 8; *p* = 0.015) (Fig. 3e). Of note, no differences were seen in activated astroglia (Null: 7.57% ± 2.77%, *n* = 8; sIL21R: 5.79% ± 3.19%, *n* = 8; *p* = 0.253) (Fig. 3f).

### Preventive Blockade of IL-21 Modifies the Immune Profile

**Fig. 4:**
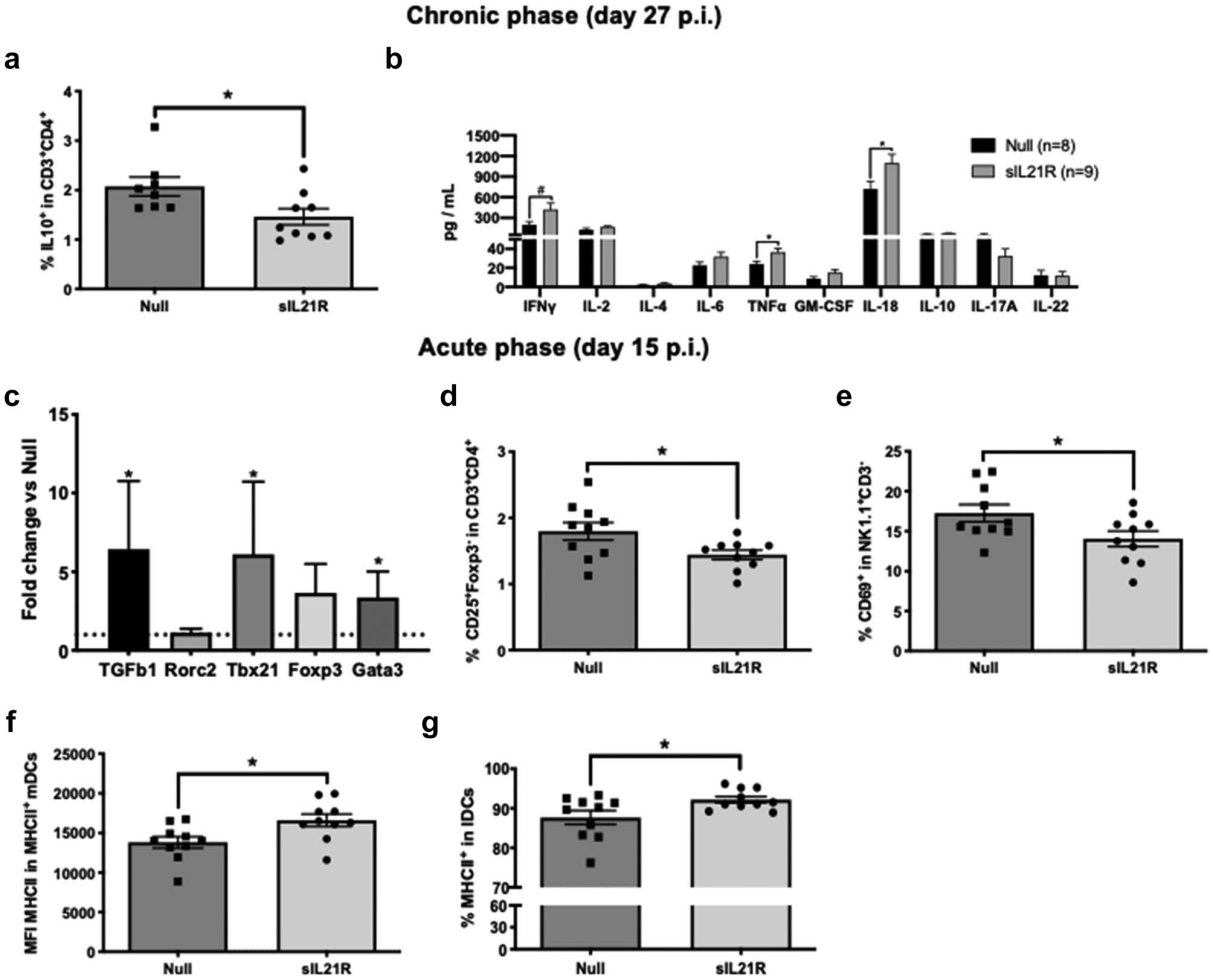
Preventive treatment with AAV8.CMV.sIL21R modifies the immunological profile of mice with EAE. In the chronic phase, sIL21R-treatment (**a**) induced a peripheral reduction in IL-10 producing CD4 T cells (CD3^+^CD4^+^IL10^+^) and (**b**) an increase in the levels of TNFα, IL-18 and a trend towards increased IFNγ in the supernatant of splenocytes stimulated with MOG_40-55_. (Null, *n* = 8; sIL21R, *n* = 9). In the acute phase, sIL21R-treatment (**c**) increased the mRNA expression of TGFb1, Tbx21 and Gata3, relative to Null group (Null, *n* = 8; sIL21R, *n* = 7). **d** The blockade of IL-21 also produced a peripheral reduction in the frequency of activated CD4 T-cells (CD25^+^Foxp3^−^ in CD3^+^CD4^+^) and **e** activated NK cells (CD69^+^ in NK1.1^+^CD3^−^) during the acute phase of EAE. However, **f** sIL21R promoted an increase in the activation of DCs which could be observed in a higher MFI of MHCII in mDCs (CD11b^+^B220^−^CD11c^high^CD8^−^) and **g** in a higher frequency of MHCII^+^ lDCs (CD11b^+^B220^−^CD11c^high^CD8^+^MHCII^+^). (Null, *n* = 10; sIL21R, *n* = 10). Data are represented as mean ± SEM (**a**, **b**, **d**, **e**, **f**, **g**) or as mean ± SD (**c**). ^#^*p* < 0.06, *p < 0.05

Once the protective effect of the treatment was observed, we decided to study the peripheral immune response in the chronic phase by analyzing various cell populations by flow cytometry as well as the cytokine expression profile of splenocytes. sIL21R-treated mice showed a lower percentage of IL-10 producing CD4 T cells (IL-10^+^ in CD3^+^CD4^+^) (Null: 2.07% ± 0.54%, *n* = 8; sIL21R: 1.46% ± 0.49%, *n* = 9; *p* = 0.028) (Fig. 4a), but no significant differences were found in the rest of analyzed immune populations (Supplementary Table 1). In addition, sIL21R preventive treatment increased the concentration of TNFα (Null: 23.99 pg/mL ± 8.44 pg/mL, *n* = 8; sIL21R: 36.36 pg/mL ± 12.47 pg/mL, *n* = 9; *p* = 0.032), IL-18 (Null: 719.20 pg/mL ± 315.10 pg/mL, *n* = 8; sIL21R: 1095.00 pg/mL ± 387.90 pg/mL, *n* = 9; *p* = 0.046) and tended to increase IFNγ (Null: 196.70 pg/mL ± 136.20 pg/mL, *n* = 8; sIL21R: 418.00 pg/mL ± 287.10 pg/mL, *n* = 9; *p* = 0.060) in the supernatants of the splenocytes stimulated with MOG_40-55_ (Fig. 4b).

Subsequently, we decided to further investigate the peripheral immunological response in sIL21R-treated mice. For this purpose, new experiments were carried out in which the mice were euthanized in the acute phase of the disease (day 15 p.i), when the immune response is already stablished. First, the expression of characteristic transcription factors and cytokines involved in EAE pathogenesis were analyzed in splenocytes by qPCR. sIL21R-treated mice showed a higher mRNA expression of *Tgfb1* (6.44 ± 4.33, *n* = 7; *p* = 0.024), *Tbx21* (Th1 cells) (6.11 ± 4.61, *n* = 7; *p* = 0.028) and *Gata3* (Th2 cells) (3.37 ± 1.65, *n* = 7; *p* = 0.043) and a trend towards increased expression of *Foxp3* (Treg cells) (3.66 ± 1.84, *n* = 7; *p* = 0.092) when results are normalized with respect to Null group. No expression change was observed in *Rorc2* (Th17 cells) (1.13 ± 0.25, *n* = 7; *p* = 0.467) (Fig. 4c). However, the expression of several genes could not be detected (*Il17a*, *Il4*, *Il10*, and *Ifng*). Interestingly, this effect was no longer observed when analyzing the expression in splenocytes from animals euthanized in the chronic phase (day 27 p.i), when the immune response is not so activated (data not shown). Thus, in this experimental model, the effect of the sIL21R treatment mainly happens during the establishment of immune response at the periphery.

sIL21R-treated mice exhibited a significantly lower percentage of activated CD4 T cells (CD25^+^Foxp3^−^ in CD3^+^CD4^+^) (Null: 1.80% ± 0.42%, *n* = 10; sIL21R: 1.44% ± 0.22%, *n* = 10; *p* = 0.029) (Fig. 4d) and a reduction in the percentage of activated NK cells (CD69^+^ in NK1.1^+^CD3^−^) (Null: 17.28% ± 3.40%, *n* = 10; sIL21R: 14.07% ± 3.08%, *n* = 10; *p* = 0.040) (Fig. 4e). On the other hand, in this acute clinical phase, the sIL21R treatment induced a greater expression of the activation marker MHCII in myeloid DCs (mDCs: CD11b^+^B220^−^CD11c^high^CD8^−^) (Null: 13,816.00 median fluorescence intensity (MFI) ± 2265.00 MFI, *n* = 10; sIL21R: 16,603.00 MFI ± 2467.00 MFI, *n* = 10; *p* = 0.017) (Fig. 4f). The sIL21R-treated group also exhibited a significantly higher percentage of MHCII^+^ lymphoid DCs (lDCs: CD11b^+^B220^−^CD11c^high^CD8^+^) (Null: 87.67% ± 5.51%, *n* = 10; sIL21R: 92.19% ± 2.57%, *n* = 10; *p* = 0.031) (Fig. 4g). No significant differences were found in the other immune populations analyzed (Supplementary Table 2) or the cytokine secretion profile (Fig. S1).

### Early Therapeutic Approach with AAVs of sIL21R-Constitutive Expression Produces a Clinical Worsening of EAE

The next step consisted in testing the effect of blocking IL-21 with a constitutive expression of AAVs in a therapeutic approach. In previous studies, we showed a decrease in the vector expression if it was administered after the induction of EAE (days 5 and 9 p.i) [25].

Based on this study, we decided to clone the therapeutic gene under the control of the CAG promoter and to double the viral dose administered in the preventive approach to ensure that the transgene was expressed at high levels when the disease was established.

**Fig. 5:**
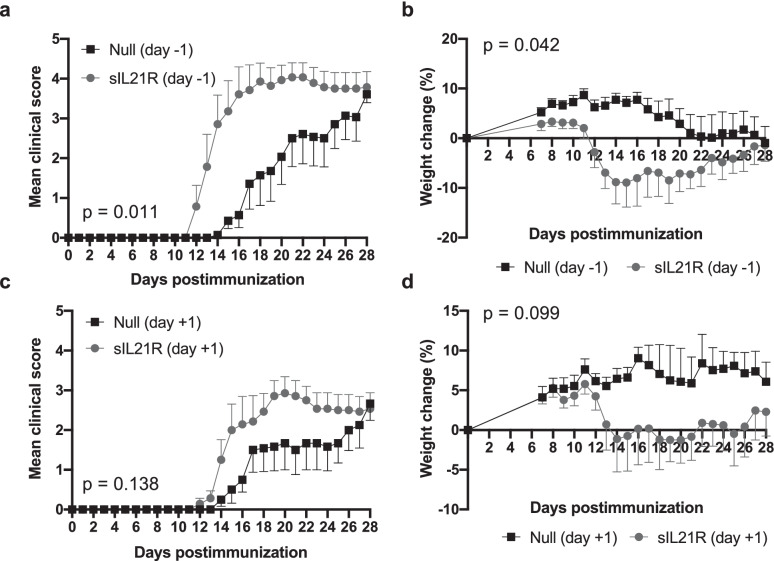
The early therapeutic treatment with AAV8.CAG.sIL21R produces a clinical worsening of EAE. Mice were immunized with MOG_40-55_ and treated with 10.^12^ vg/mouse of AAV8.CAG.sIL21R or AAV8.CAG.Null the day before (day −1) or the day after (day +1) the induction of EAE. **a** Mean daily clinical score of EAE-incident mice treated at day −1. **b** Average daily weight change of incident animals treated at day −1 relative to initial weights on the day of EAE induction. **c** Mean daily clinical score of incident animals treated at day +1. **d** Average daily weight change of incident animals treated at day +1 relative to initial weights on the day of EAE induction. Data were obtained from a single experiment (Null (day −1): *n* = 7; sIL21R (day −1): *n* = 7; Null (day +1): *n* = 6; sIL21R (day +1): *n* = 7). Data are represented as mean ± SEM. *p < 0.05

Furthermore, considering that an AAV requires between 1 and 2 weeks to reach maximum expression [27, 28], we decided to perform an early therapeutic approach in which 10^12^ vg per animal of AAV8.CAG.sIL21R or AAV8.CAG.Null were administrated the day before (day −1) or after (day +1) the immunization day. Surprisingly, when the clinical variables of the EAE-incident mice were analyzed, sIL21R-treated group developed a clinical worsening of the disease. Although differences reached statistical significance in the group administered on day −1 (mean clinical score AUC (Null: 28.63 ± 15.95, *n* = 7; sIL21R: 56.54 ± 20.16, *n* = 7; *p* = 0.011), weight change AUC (Null: 90.58 ± 123.20, *n* = 7; sIL21R: -83.73 ± 161.50, *n* = 7; *p* = 0.042)) (Fig. 5a, b), the experimental group administered on day +1 also showed a trend towards clinical worsening (mean clinical score AUC (Null: 21.33 ± 15.70, *n* = 6; sIL21R: 35.70 ± 13.03, *n* = 7; *p* = 0.138), weight change AUC (Null: 142.10 ± 79.28, *n* = 6; sIL21R: 25.89 ± 139.00, *n* = 7; *p* = 0.099)) (Fig. 5c, d).

**Fig. 6:**
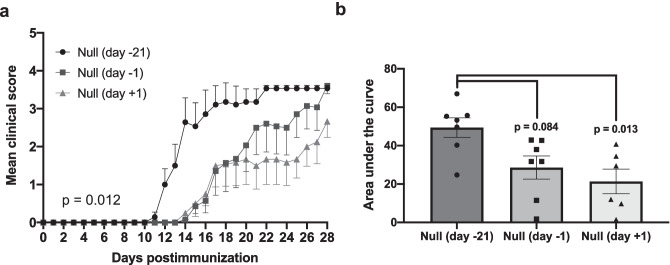
The administration of AAV vectors close to the induction of EAE produces a reduction in clinical severity. Mice were immunized with MOG_40-55_ and treated with 10^12^ vg/mouse of AAV8.CAG.Null 21 days before (day −21), the day before (day −1) or the day after (day +1) the induction of EAE. **a** Mean daily clinical score of EAE-incident mice of all groups. **b** Null-treated mice at day −1 and day +1 developed a milder disease compared with Null group preventively treated at day −21. Data were obtained from a single experiment (Null (day −21): *n* = 7; Null (day −1): *n* = 7; Null (day +1): *n* = 6). Data are represented as mean ± SEM

When the clinical data of this experiment were analyzed, it was observed that the severity of disease, both in the sIL21R and the Null-treated groups, seemed to become milder when the AAVs were administered at day +1 compared to the group administered on day −1, so we hypothesized that the administration of AAVs on days close to immunization produced an alteration in the clinical course. To confirm this hypothesis, Null groups included in the early therapeutic experiment were compared with a Null group administered in preventive (day −21 p.i) of an experiment carried out in parallel. When analyzing the clinical variables of EAE-incident mice, we observed that the clinical severity was significantly lower when vectors were administered close to the day of immunization (Null day −21: 49.41 ± 13.48, *n* = 7; Null day −1: 28.63 ± 15.95, *n* = 7; Null day +1: 21.33 ± 15.70, *n* = 6; *p* = 0.012) (Fig. 6a, b). However, no statistically significant differences were found when weight change was analyzed (Null day −21: 64.05 ± 119.60, *n* = 7; Null day −1: 90.58 ± 123.20, *n* = 7; Null day +1: 142.10 ± 79.28, *n* = 6; *p* = 0.457).

### Treatment with AAV8.TetOn.sIL21R Improves Established Symptoms of EAE

To test the therapeutic potential of sIL21R in the absence of viral capsid effect, Dox-inducible AAVs were developed. This vectors can be administered prior to the induction of EAE and induce the expression of the therapeutic protein with the appearance of the first neurological signs of the disease. In a previous pilot study, the experimental conditions of 10^12^ vg per animal and a Dox dose of 25 mg/kg were selected as those that reported acceptable liver expression in animals (data not shown).

**Fig. 7:**
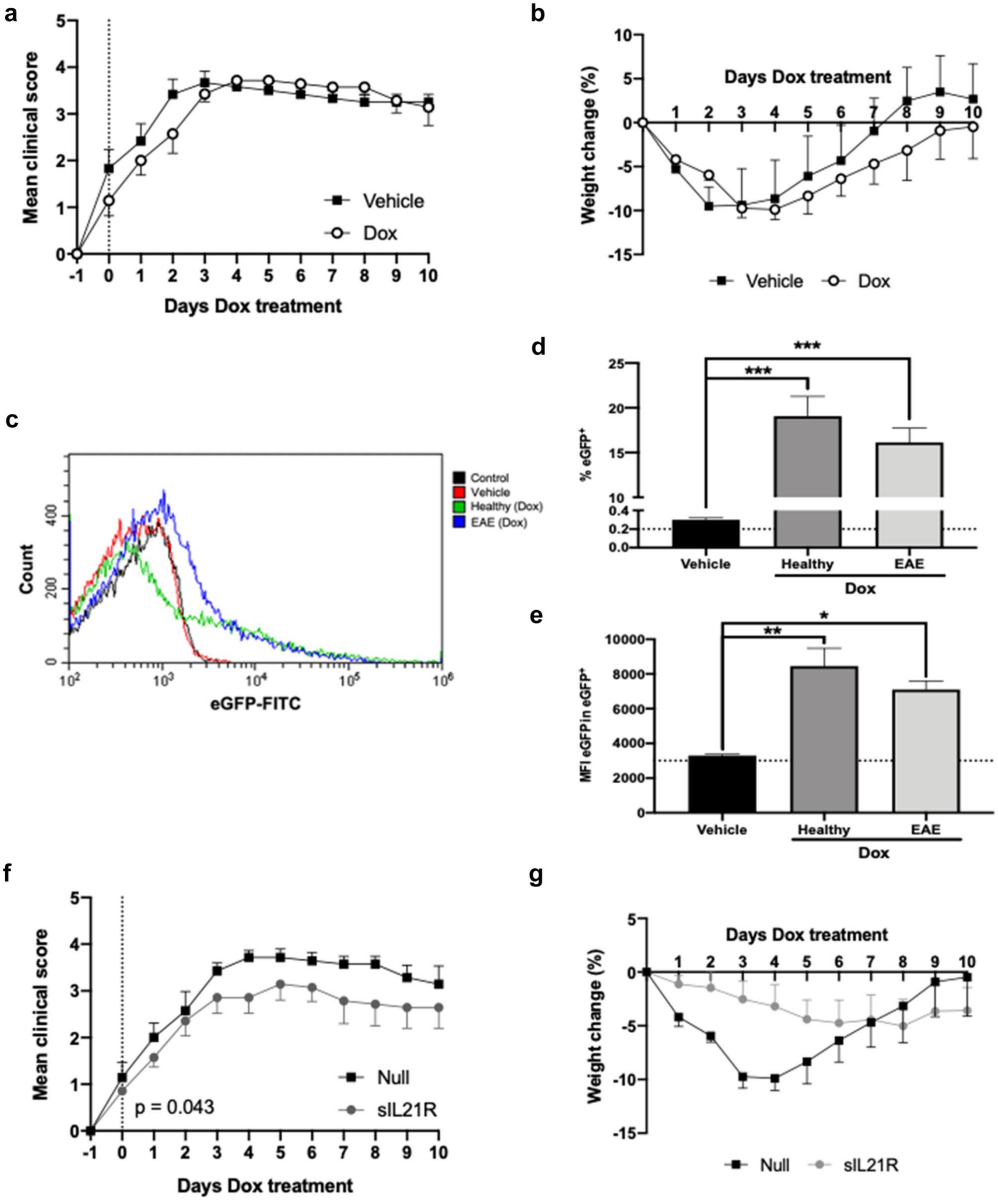
Dox administration does not alter the clinical course and the treatment with AAV8.TetOn.sIL21R improved established symptoms of EAE. Mice were immunized with MOG_40-55_, treated with 10^12^ vg/mouse of AAV8.TetOn.Null, AAV8.TetOn.eGFP or AAV8.TetOn.sIL21R 14 days before the induction of EAE, and transgene expression was induced by daily i.p. administration of 25 mg/kg doxycycline (Dox) from the day of onset of neurological symptoms. **a** Mean daily clinical score of EAE mice treated with vehicle or Dox. **b** Average daily weight change of EAE animals treated with vehicle or Dox relative to weight prior to onset of neurological symptoms. Day 0 is considered the first day of Dox administration. Data were obtained from a single experiment (Vehicle: *n* = 6; Dox: *n* = 7). **c** Representative flow cytometry histogram of eGFP^+^ hepatocytes of the different experimental groups. **d** Frequency and **e** MFI of eGFP.^+^ hepatocytes obtained from healthy and EAE mice treated with AAV8.TetOn.eGFP. Baseline values were provided by control animals without vector and without Dox (*n* = 2). Data were obtained from a single experiment (Vehicle: *n* = 3; Dox (Healthy: *n* = 8; EAE: *n* = 8)). **f** Mean daily clinical score of mice administered with AAV8.TetOn.Null or AAV8.TetOn.sIL21R vectors and treated with Dox. **g** Average daily weight change of EAE-incident mice administered with AAV8.TetOn.Null or AAV8.TetOn.sIL21R vectors and treated with Dox relative to weight prior to onset of neurological symptoms. Day 0 was considered the first day of Dox administration. Data were obtained from a single experiment (Null: *n* = 7; sIL21R: *n* = 7). Data are represented as mean ± SEM. *p < 0.05, **p < 0.01, ***p < 0.001

The use of a Dox-inducible system forced us to consider the influence that the inductor could have on the clinical course and its toxicity in the animal model. Thus, comparing both the mean clinical score (*p* = 0.559) and the percentage of variation in weight (*p* = 0.611) between EAE animals treated with Dox and the vehicle (saline solution), no differences were observed (Fig. 7a, b). In addition, EAE mice presented hepatic eGFP expression levels similar to those presented by healthy control animals both in the percentage of eGFP^+^ cells (Vehicle: 0.30% ± 0.04%, *n* = 3; Healthy: 19.07% ± 6.23%, *n* = 8; EAE: 16.15% ± 4.50%, *n* = 8) (Fig. 7d) and the level of eGFP expression (Vehicle: 3297 MFI ± 135.50 MFI, *n* = 3; Healthy: 8459 MFI ± 2890 MFI, *n* = 8; EAE: 7100 MFI ± 1392 MFI, *n* = 8) (Fig. 7e). These results validated the use of these inducible vectors for therapeutic approaches in this animal model of MS.

Finally, mice administered with AAV8.TetOn.sIL21R showed less severe symptoms once the expression of sIL21R was induced compared to the Null-control group (Null: 31.64 ± 2.82, *n* = 7; sIL21R: 25.75 ± 7.46, *n* = 7; *p* = 0.043) (Fig. 7f). No statistically significant differences were found in the AUC of weight change (Null: −5 1.38 ± 41.47, *n* = 7; sIL21R: −31.77 ± 39.83, *n* = 7; *p* = 0.384) (Fig. 7g).

## Discussion

The pleiotropy and the wide range of action of IL-21 on the components of the immune system have placed this cytokine in the central axis of various pathologies such as cancer and immune-mediated diseases like MS, RA, SLE, or type 1 diabetes [29]. In the present study, we have developed a gene therapy strategy for blocking IL-21 signalling using viral vectors encoding a sIL21R and have tested it in a mouse model of EAE. In in vitro studies, sIL21R exhibited the desired characteristics of solubility and secretability, as well as decreased IL-21 signalling when incubated in the presence of sIL21R in murine splenocytes.

First, we tested the prophylactic potential of sIL21R by administering constitutively expressed AAV8 vectors in EAE animals. Since AAV vectors require at least 2 weeks to reach the maximum level of transgene expression [27, 28], the preventive in vivo experiments involved the administration of the viral vectors 3 weeks prior to mice immunization (day −21 p.i). Vectors were systemically administered by i.v. injection since the origin of the autoimmune response in the EAE model is in the periphery [30, 31]. After carrying out two independent experiments, a clinical improvement was observed in the sIL21R-treated group compared to the control Null-treated group. This improvement was caused by the 40% reduction in the EAE incidence of sIL21R-treated mice. These results were confirmed by histopathological analysis, in which sIL21R-treated mice showed significantly less CD3 T cell infiltration, demyelination, microglial activation, and axonal damage in the CNS. Our results were in line with a study in the EAE model where daily exogenous administration of IL-21 during 7 days prior to immunization triggered a more severe clinical outcome [32]. Similar results were found in a study in which knockout (KO) mice for IL-21 (IL-21^−/−^) developed milder EAE [33]. In addition, a more recent study in the spontaneous EAE 2D2 IL-21R^−/−^ mice determined that IL-21 signalling is critical for the development of the pathology [34]. However, some studies in IL-21^−/−^ and IL-21R^−/−^ mice have showed contradictory results [35, 36]. Thus, there are studies showing that the absence of the IL-21 signalling induces the development of more severe clinical signs. Due to it, these contradictory results may be explained by the different genetic backgrounds of the transgenic animals, differences in immunization protocols and experimental approaches applied. In addition, it is worth mentioning that KO animals for elements of the immune system eventually develop compensatory immune mechanisms capable of replacing the functions of the missing protein [37, 38]. This highlights the importance of achieving homogeneous experimental designs for comparative purposes, especially for strategies involving pleiotropic cytokines such as IL-21.

Subsequently, immunological studies were performed on splenocytes in the chronic phase (day 27 p.i) and further extended in the acute phase (day 15 p.i) of the experimental disease. In the chronic phase, the sIL21R-treated group showed a reduction in the number of IL-10^+^ CD4 T cells in the periphery, contrary to expected. However, this effect could not be seen during the acute phase. In preventive approaches, the reduction of anti-inflammatory pathways, such as those mediated by Treg cells, is a common occurrence. In a recent study, it was observed that mice that had been protected from EAE showed a reduction in the frequency of Treg cell in the periphery [39] whereas in another study this phenomenon is justified by the migration of these suppressor cells into the CNS [40]. In our case, we hypothesized that the sIL21R-treated mice experienced a lower initial inflammatory response, which required a lower compensatory response mediated by IL-10. In this clinical stage, we also observed an increase in TNFα and IL-18, and a trend towards increased IFNγ levels in the supernatant of ex vivo MOG-stimulated splenocytes. Interestingly, increased levels of TNFα and IFNγ cannot be considered as a univocally negative effect of the treatment as these molecules have been associated to controversial roles in EAE and MS. Anti-TNFα treatments were found to be effective in the passive transfer-induced EAE model, contrary to the obtained results by active induction [41, 42]. Even exogenous administration of TNFα to TNFα KO mice rescued them from further severe EAE, while clinical trials in MS patients treated with a soluble TNFα receptor (Lenercept) resulted in clinical worsening and had to be discontinued [43, 44]. On the other hand, although passive transfer of IFNγ-producing autoreactive Th1 cells can induce EAE [45], anti-IFNγ treatments cause an exacerbation of clinical symptoms [46–48] and exogenous administration of IFNγ generates a protective effect in EAE [47, 48]. In contrast, the administration of IFNγ in MS patients results in an exacerbation of disease [49]. Furthermore, IFNγ has been shown to inhibit the development of Th17 cells [50], although we did not observed such effect.

In the acute phase, sIL21R-treated mice showed increased gene expression of TGF*β*-1 in the periphery, a potent tolerance inducer whose main function is to reduce CD4 T cell activation in this clinical stage [51, 52]. This would explain the reduction in the percentage of activated CD4 T cells observed in the periphery. It is also widely accepted that TGF*β* is a key molecule in the balance of the Th17/Treg response, dependent on the presence of IL-6, but also IL-21 [53]. Although the treatment produced a trend towards increased *FoxP3* expression in the periphery, we could not observe an alteration in the frequency of Treg or Th17 cells by flow cytometry as well nor in the gene expression of RORγ t. Although IL-21 is able to induce differentiation towards Th17 in the presence of TGF*β*, this function can be effectively compensated by the presence of IL-6 [35]. This could explain why IL-21 blockade in monotherapy does not produce an effective regulation of this immune population. This idea is supported by a previous study that combined a treatment of IL-6 and IL-21 blockade in the collagen-induced RA model triggering a synergistic effect and significantly decreasing the number of Th17 cells [54]. Our gene expression studies also showed an increase in Gata3, a characteristic factor of the Th2 cell population, but this result was not supported by an increase either in the percentage of IL-4^+^ T cells or in the levels of IL-4 in the supernatant of MOG_40-55_ stimulated splenocytes. This could be due to a delay between the increase in mRNA expression and the increase of activation and synthesis of cytokines characteristic of this cellular pathway. We also observed a reduction in the number of activated NK cells in the periphery of sIL21R-treated mice in the acute phase, which would evidence that IL-21 activity was reduced by the treatment since this cytokine has a stimulatory effect on NK cell activation, cytotoxicity and IFNγ production [55].

Mice treated with sIL21R also showed increased peripheral activation of DCs in the acute phase, which is in line with the capacity of IL-21 to inhibit the maturation and activation of DCs by reducing surface expression of MHCII, CD80 and CD86 and TNFα secretion [56, 57]. This DC activation status could be related with the increased expression of Tbx21, the main Th1 cell transcription factor, in this clinical stage.

The next step was to study the effect of constitutive sIL21R expression in a therapeutic approach using AAV8 vectors. Previous results from our group showed that transgene expression was significantly lower when viral vectors were administered after immunization [25]. Therefore, we increased hepatic expression of the viral vectors by using the CAG promoter as well as administered a double viral dose to the animals. We administered the vectors close to the induction of EAE (days −1 and +1 p.i) to synchronize the time of maximum transgene expression with the onset of clinical symptomatology. In contrast to the results obtained in the prophylactic approach, the sIL21R-treated incident mice developed the disease earlier than control-treated mice and the clinical course was more severe. This result was in line with another study in a relapsing–remitting model of EAE in which IL21R-Fc was administered 2 days before immunization and also revealed a worsening of the clinical outcome [58]. Bioavailability of a specific cytokine and the relative abundance of its receptor in target cells is frequently associated with the variation of the cytokine functionality. This is the case of IL-2, an essential cytokine for Treg cell functionality and survival [59], since its partial blockade increases the expansion and suppressive function of this cell type [60]. Taking this into account and since AAV vectors can initiate their expression within few days after administration [61], we believed that AAV administration close to EAE induction lead to a partial blockade of IL-21 that resulted in a potentiation effect of the pro-inflammatory function of the cytokine.

Furthermore, the reduction in clinical severity observed in the Null-treated groups administered close to the immunization day compared to the Null-treated group administered 21 days before EAE immunization, led us to hypothesize that an immune response induced by the viral capsid likely reduces the response against the encephalitogenic peptide. An additional hypothesis that could explain this result is the increase in cortisol levels in the mice generated by the administration of the viral vectors itself, as such stress could also lead to a reduction in clinical severity. To address this issue, we designed a therapeutic approach where we administered a Dox-inducible system, AAV8.TetOn.sIL21R, 14 days before EAE induction and we induced Dox-mediated transgene expression at the onset of the neurological signs.

To study the effect of Dox in the onset and progression of EAE, we analysed the clinical course and weight loss of the animals and found no differences. Furthermore, by assessing the eGFP hepatic expression, we concluded that the inducible system worked efficiently in the EAE model, validating its usefulness for future AAV-mediated therapeutic approaches in this model. Interestingly, mice administered with AAV8.TetOn.sIL21R developed less severe clinical signs than the control group. However, it seems that the contribution of IL-21 once the clinical symptoms are established is not as relevant as its contribution in the initiation of the immune response. Thus, although the highest levels of IL-21 are produced during the onset of the immune response and in the acute phase [36], the exogenous administration of IL-21 from day 9 p.i does not result in an appreciable worsening of the clinical course [32]. On the other hand, IL-21 blockade by therapeutic administration of IL21R-Fc has been effective in other animal models of RA [62] or SLE [63], producing a reduction in clinical severity and an increase in the survival rate. Immunological studies should be carried out to explain the clinical improvement observed in this approach. In addition, it would also be interesting to study the effect of the sIL21R-inducible vectors in the different inflammatory phases in a relapsing–remitting EAE model.

To sum up, although further clinical and immunological studies are needed to define the suitable therapeutic time window for sIL21R treatment, this gene therapy strategy has shed light on the pathogenic role of IL-21 in the different clinical and immunological stages of EAE. It may represent a breakthrough in the current MS therapeutic landscape where most treatments involve the use of antibodies and recombinant proteins. Since current therapies involve long-term routine administration, which explains the large financial outlay, and the ability to respond to these treatments declines as the disease progresses [64], making the effectiveness-safety ratio less favorable over time. AAV gene therapy treatments might be a great promise for the long-term treatment of MS.

## Supplementary Information

Below is the link to the electronic supplementary material.

Supplementary file1 (DOCX 63 kb)

Supplementary file2 (PDF 3133 kb)

Supplementary file3 (PDF 279 kb)

Supplementary file4 (PDF 272 kb)

Supplementary file5 (PDF 496 kb)

Supplementary file6 (PDF 8993 kb)

Supplementary file7 (PDF 4902 kb)

## Abbreviations

AAV: Adeno-associated vector
Ad5: Adenovirus 5
AUC: Area under curve
CNS: Central nervous system
DCs: Dendritic cells
Dox: Doxycycline
EAE: Experimental autoinmune encephalomyelitis
GFAP: Glial fibrillary acidic protein
i.p.: Intraperitoneal
i.v.: Intravenous
IL-21: Interleukin 21
IL-21R: Interleukin 21 receptor
KO: Knockout
LEA: *lycopersicon esculentum* Lectin
MBP: Myelin basic protein
MFI: Median fluorescence intensity
MOI: Multiplicity of infection
MS: Multiple sclerosis
NK: Natural killer
p.i: Postimmunization
RA: Rheumatoid arthritis
SLE: Systemic lupus erythematosus
SD: Standard deviation of the mean
SEM: Standard error of the mean
sIL21R: Soluble interleukin 21 receptor
Treg: T regulatory
vg: Viral genomes
