# Supplementary material for: Therapeutic Effect of IL-21 Blockage by Gene Therapy in Experimental Autoimmune Encephalomyelitis
Source: Neurotherapeutics. 2022 Jul 28;19(5):1617–33. doi: 10.1007/s13311-022-01279-8 (PMC9606180; doi:10.1007/s13311-022-01279-8)
Supplement: Supplementary file 1 — Supplementary file1 (DOCX 63 kb) [file 13311_2022_1279_MOESM1_ESM.docx]

**SUPPLEMENTARY TABLES**

**Flow cytometry data from splenocytes obtained in chronic phase**

| **Panel** | **Immune population** | **Markers** | **Null (n=9)** | **sIL21R (n=8)** |
| --- | --- | --- | --- | --- |
| **T cells** | **T cells** | CD3^+^ | 68.20% ± 6.54% | 60.59% ± 10.41 % |
|  | **CD4 T cells** | CD4^+^ in T cells | 80.96% ± 2.74% | 70.00% ± 5.16% |
|  | **CD8 T cells** | CD8^+^ in T cells | 15.02% ± 2.20% | 16.99% ± 5.06% |
|  | **CD4 Treg cells** | CD25^+^ Foxp3^+^ in CD4 T cells | 18.25% ± 3.89% | 16.94% ± 2.34% |
|  | **CD8 T reg cells^(*)^** | CD25^+^ Foxp3^+^ in CD8 T cells | No valuable | No valuable |
|  | **Activated CD4 T cells^(*)^** | CD25^+^Foxp3^-^ in CD4 T cells | No valuable | No valuable |
| **Pro-inflammatory cytokines T cells producers** | **IL17 CD4 T cells** | IL17^+^ in CD4 T cells | 0.89% ± 0.17% | 0.79% ± 0.16% |
|  | **IL17 CD8 T cells^(*)^** | IL17^+^ in CD8 T cells | No valuable | No valuable |
|  | **IFNg CD4 T cells** | IFNg^+^ in CD4 T cells | 8.27% ± 2.09% | 8.54% ± 1.55% |
|  | **IFNg CD8 T cells** | IFNg^+^ in CD8 T cells | 17.38% ± 4.40% | 18.06% ± 3.85% |
| **Anti-inflammatory of regulatory cytokines T cells producers** | **IL4 CD4 T cells** | IL4^+^ in CD4 T cells | 1.75% ± 0.40% | 1.79% ± 0.37% |
|  | **IL4 CD8 T cells^(*)^** | IL4^+^ in CD8 T cells | No valuable | No valuable |
|  | **IL10 CD8 T cells^(*)^** | IL10^+^ in CD8 T cells | No valuable | No valuable |

**^(*)^** No valuable populations due to a low number of representative events (<100)

**Supplementary table 1.** Values obtained for the rest of immune populations analyzed by flow cytometry in splenocytes isolated in chronic phase (day 27 p.i.) from mice preventively treated with sIL21R. Data are presented as mean ± SD.

**Flow cytometry data from splenocytes obtained in acute phase**

| **Panel** | **Immune population** | **Markers** | **Null (n=10)** | **sIL21R (n=10)** |
| --- | --- | --- | --- | --- |
| **T cells** | **T cells** | CD3^+^ | 16.61% ± 6.48% | 21.52% ± 9.26 % |
|  | **CD4 T cells** | CD4^+^ in T cells | 75.07% ± 4.22% | 76.05% ± 4.01% |
|  | **CD8 T cells** | CD8^+^ in T cells | 17.52% ± 4.62% | 17.73% ± 4.91% |
|  | **CD4 Treg cells** | CD25^+^ Foxp3^+^ in CD4 T cells | 13.30% ± 3.44% | 13.48% ± 3.74% |
|  | **CD8 T reg cells^(*)^** | CD25^+^ Foxp3^+^ in CD8 T cells | No valuable | No valuable |
| **Pro-inflammatory cytokines T cells producers** | **IL17 CD4 T cells** | IL17^+^ in CD4 T cells | 1.52% ± 0.58% | 1.48% ± 0.63% |
|  | **IL17 CD8 T cells^(*)^** | IL17^+^ in CD8 T cells | No valuable | No valuable |
|  | **IFNg CD4 T cells** | IFNg^+^ in CD4 T cells | 11.78% ± 4.08% | 11.24% ± 3.87% |
|  | **IFNg CD8 T cells** | IFNg^+^ in CD8 T cells | 36.51% ± 6.55% | 40.07% ± 5.93% |
| **Anti-inflammatory of regulatory cytokines T cells producers** | **IL4 CD4 T cells** | IL4^+^ in CD4 T cells | 0.27% ± 0.11% | 0.27% ± 0.11% |
|  | **IL4 CD8 T cells^(*)^** | IL4^+^ in CD8 T cells | No valuable | No valuable |
|  | **IL10 CD4 T cells** | IL10^+^ in CD4 T cells | 0.78% ± 0.19% | 0.71% ± 0.16% |
|  | **IL10 CD8 T cells^(*)^** | IL10^+^ in CD8 T cells | No valuable | No valuable |
| **Macrophages** | **Macrophages** | Ly6G^-^F4/80^+^CD11b^+^ | 10.81% ± 4.69% | 9.78% ± 6.39% |
|  | **M2 Macrophages^(*)^** | Ly6C^-^ CD206^+^ in Macrophages | No valuable | No valuable |
| **NK cells** | **NK cells** | NK1.1^+^ CD3^-^ | 2.39% ± 0.77% | 2.93% ± 0.82% |
|  | **NKT cells** | NK1.1^+^ CD3^+^ | 1.07% ± 0.38% | 0.98% ± 0.24% |
|  | **Activated NKT cells** | CD69^+^ in NKT cells | 21.68% ± 5.61% | 19.02% ± 2.15% |
| **B cells** | **B cells** | B220^+^ | 72.73% ± 10.30% | 72.81% ± 9.98% |
|  | **Activated B cells** | MHCII^+^ in B cells | 96.29% ± 6.17% | 98.04% ± 0.89% |
|  | **Breg cells** | CD1d^high^ CD5^+^ in B cells | 1.04% ± 0.26% | 1.02% ± 0.40% |
|  | **Plasmatic cells^(*)^** | B220^-^ CD19^-^MHCII^-^ CD138+ | No valuable | No valuable |

**^(*)^** No valuable populations due to a low number of representative events (<100)

(Continue of supp. table 2)

| **Panel** | **Immune population** | **Markers** | **Null (n=10)** | **sIL21R (n=10)** |
| --- | --- | --- | --- | --- |
| **DCs** | **Myeloid cells** | CD11b^+^ B220^-^ | 55.09% ± 11.43% | 49.31% ± 15.71% |
|  | **Total DCs** | CD11c^high^ in myeloid cells | 3.04% ± 2.30% | 3.14% ± 0.89% |
|  | **Activated total DCs** | MHCII^+^ (%) in total DCs | 93.42% ± 2.77% | 94.68% ± 1.44% |
|  |  | MHCII^+^ (MFI) in total DCs | 12802 MFI ± 2258 MFI | 14686 MFI ± 1749 MFI |
|  |  | CD80^+^ (%) in total DCs | 72.80% ± 4.38% | 71.65% ± 4.46% |
|  |  | CD80^+^ (MFI) in total DCs | 4816 MFI ± 488.30 MFI | 4721 MFI ± 432.90 MFI |
|  | **mDCs** | CD8a^-^ in total DCs | 2.32% ± 1.86% | 2.28% ± 0.77% |
|  | **Activated mDCs** | MHCII^+^ (%) in mDCs | 95.06% ± 1.80% | 95.21% ± 1.19% |
|  |  | CD80^+^ (%) in mDCs | 74.98% ± 5.26% | 73.35% ± 5.17% |
|  |  | CD80^+^ (MFI) in mDCs | 5014 MFI ± 551.20 MFI | 4955 MFI ± 584.70 MFI |
|  | **lDCs** | CD8a^+^ in total DCs | 0.66% ± 0.41% | 0.80% ± 0.23% |
|  | **Activated lDCs** | MHCII^+^ (MFI) in lDCs | 9819 MFI ± 2092 MFI | 11081 MFI ± 1788 MFI |
|  |  | CD80^+^ (%) in lDCs | 48.59% ± 7.06% | 48.08% ± 4.67% |
|  |  | CD80^+^ (MFI) in lDCs | 5126 MFI ± 415.8 MFI | 4987 MFI ± 211.20 MFI |

**Supplementary table 2.** Values obtained for the rest of immune populations analyzed by flow cytometry in splenocytes isolated in acute phase (day 15 p.i.) from mice preventively treated with sIL21R. (Null: n = 10; sIL21R: n = 10). Data are presented as mean ± SD.

**SUPPLEMENTARY FIGURES**

**Fig. S1** Concentration of cytokines detected in the SN of MOG_40-55_-stimulated splenocytes in acute phase (day 15 p.i.) from animals preventively treated with sIL21R. (Null: n = 8; sIL21R: n = 8). Data are represented as mean ± SEM.
